# A Rare Complication of Hyperplastic Gastric Polyp

**DOI:** 10.1155/2013/631975

**Published:** 2013-01-20

**Authors:** Suresh Kumar Nayudu, Masooma Niazi, Bhavna Balar, Kavitha Kumbum

**Affiliations:** ^1^Division of Gastroenterology, Bronx-Lebanon Hospital Center, Albert Einstein College of Medicine, Yeshiva University, Bronx, NY 10461, USA; ^2^Department of Medicine, Bronx-Lebanon Hospital Center, Albert Einstein College of Medicine, Yeshiva University, Bronx, NY 10461, USA; ^3^Department of Pathology, Bronx-Lebanon Hospital Center, Albert Einstein College of Medicine, Yeshiva University, Bronx, NY 10461, USA

## Abstract

Hyperplastic gastric polyps are incidentally diagnosed during upper gastrointestinal endoscopy. They are known to cause gastric outlet obstruction and chronic blood loss leading to iron deficiency anemia. However, hyperplastic gastric polyp presenting as acute severe upper gastrointestinal bleeding is very rare. To the best of our knowledge, there have been two cases of hyperplastic gastric polyps presenting as acute gastrointestinal bleeding in the medical literature. We present a case of a 56-year-old African American woman who was admitted to our hospital with symptomatic anemia and sepsis. The patient developed acute upper gastrointestinal bleeding during her hospital stay. She underwent emergent endoscopy, but bleeding could not be controlled. She underwent emergent laparotomy and wedge resection to control the bleeding. Biopsy of surgical specimen was reported as hyperplastic gastric polyp. We recommend that physicians should be aware of this rare serious complication of hyperplastic gastric polyps as endoscopic polypectomy has diagnostic and therapeutic benefits in preventing future complications including bleeding.

## 1. Introduction

Hyperplastic gastric polyps are the second most common variety of gastric polyps in the United States of America (USA) preceded by fundic gland polyps [1]. Hyperplastic polyps have been known to present clinically as gastric outlet obstruction or chronic occult blood loss leading to iron deficiency anemia [2, 3]. However, to the best of our knowledge, acute gastrointestinal bleeding in association with hyperplastic gastric polyps has been reported very rarely [3, 4]. We present a case of a 56-year-old African American woman with acute severe upper gastrointestinal bleeding which could not be controlled with endoscopic measures and finally underwent surgical intervention to control the bleeding.

## 2. Case Presentation

A 56-year-old African American woman was brought to the emergency room (ER) by her family members with generalized weakness of 3 weeks duration. She also reported generalized body aches, subjective fever, stiffness of joints in the morning, skin rash, and loss of appetite. She denied any abdominal pain, nausea, vomiting, diarrhea, constipation, or urinary symptoms. She has known medical history of rheumatoid arthritis and pernicious anemia. She denied any major surgical procedures in the past or allergies to any medications. Her current medications included methotrexate, celecoxib, and folic acid. She never used tobacco, alcohol, or recreational drugs. As per family, the patient has been depressed, not eating well, and noncompliant to medical care for few weeks.

On initial evaluation in ER she was found to be hypotensive and afebrile. Her physical exam revealed skin scars on chest wall consistent with history of accidental burns in the past and stage 3 sacral ulcers. Her respiratory, gastrointestinal, and neurological examination did not reveal any gross abnormalities. Her blood pressure responded to fluids and the patient appeared to be stable. Her initial labs revealed anemia with hemoglobin of 6.4 grams/dL, elevated prothrombin time of 18.6, elevated blood urea nitrogen (BUN) of 40 milligrams/dL, low albumin level of 2.1 grams/dL, alanine aminotransferase 39 units/liter, aspartate aminotransferase of 107 units/liter, and elevated creatinine kinase of 750 units/liter.

She was admitted to the medical intensive care unit (MICU) for work up and management of severe symptomatic anemia and hypotension. She received multiple packed red blood cell (PRBC) transfusions. Her hemoglobin and hematocrit improved proportionately with the amount of PRBC transfusions. Her blood and urine cultures were tested positive for gram negative bacilli, subsequently identified as *Escherichia coli* for which she received broad spectrum antibiotics. After extensive work up as advised by rheumatology and hematology teams, anemia was deemed secondary to methotrexate induced bone marrow suppression. She was also evaluated by psychiatry and recommended medical management for depression. At the end of the first week of her hospital stay she was transferred to the step down unit, followed by general medical floor, and subsequently planned for discharge. 

On 10th day of her hospital stay, patient suddenly became hypotensive and lethargic. She was resuscitated with fluids but did not improve. She was placed on mechanical ventilator and vasopressor support and transferred to MICU. Fresh blood was noted on gastric lavage and rectal exam revealed melena. Her hemoglobin and hematocrit dropped significantly. She underwent emergent esophagogastroduodenoscopy (EGD) and was found to have a large amount of fresh blood in stomach and a bleeding lesion proximal to incisura angularis. Bleeding could not be controlled with endoscopic measures. She was taken to the operating room and underwent emergent laparotomy. During surgery she was found to have bleeding vascular lesion towards the lesser curvature of stomach proximal to incisura angularis. The patient underwent wedge resection and cessation of bleeding documented. She received multiple packed red blood cell transfusions during the course but did not have further bleeding.

**Figure 1:**
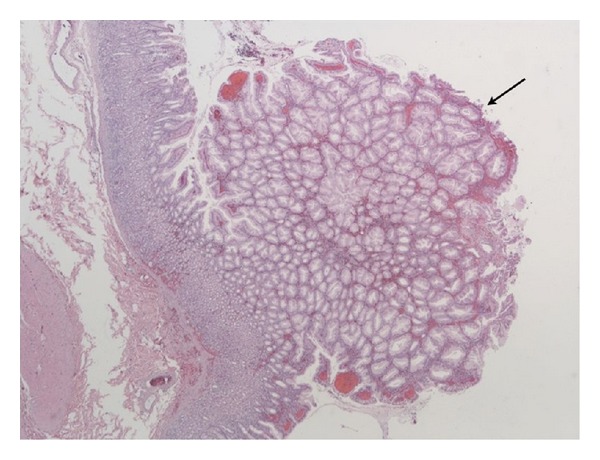
Microscopic image of surgical specimen showing hyperplastic gastric polyp.

**Figure 2:**
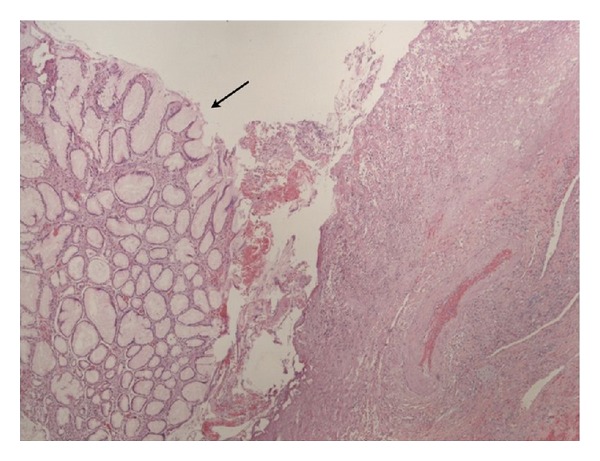
Magnified view of hyperplastic gastric polyp.

Surgical specimen was sent to pathology and was reported as a hyperplastic gastric polyp (Figures 1 and 2). She had prolonged hospital course which was complicated by recurrent sepsis and multiorgan failure. She underwent multiple procedures including tracheostomy and gastrostomy tube insertions. Her condition eventually deteriorated and she expired during the 12th week of her hospital stay.

## 3. Discussion

Gastric polyps have been reported on an average of 6% of upper gastrointestinal endoscopies [1, 5]. In USA, the majority of gastric polyps are fundic gland polyps which have been attributed to proton pump inhibitors [1, 5] but in areas where the prevalence of *Helicobacter pylori* is high, hyperplastic polyps have been reported more frequently [6–8]. However, this patient neither had any documented history of *Helicobacter pylori* infection nor use of proton pump inhibitors.

The majority of gastric polyps are found in the stomach, although they can arise rarely from ectopic gastric mucosa in other areas of gastrointestinal tract like esophagus and duodenum [9–12]. Several etiopathological mechanisms have been reported including *Helicobacter pylori*, cyclooxygenase-2 expression (COX 2), xanthelasma, and pernicious anemia [6, 8, 13, 14]. Usually hyperplastic gastric polyps do not cause any symptoms and they are diagnosed incidentally during the upper gastrointestinal endoscopy [5, 15]. However, they can rarely present with gastric outlet obstruction and iron deficiency anemia due to chronic blood loss [2, 16–18].

The most important long-term complication of hyperplastic gastric polyp is its potential to transform into a malignant tumor [19, 20]. Endoscopic polypectomy is the current recommended management for hyperplasic gastric polyps [16, 21, 22]. Testing and eradication of *Helicobacter pylori* has been recommended in all cases [6, 7].

Hyperplastic gastric polyp presenting with acute upper gastrointestinal bleeding is very rare. On our review of the literature using Pubmed database, we found only two cases of hyperplastic gastric polyps, presenting with acute upper gastrointestinal bleeding [3, 4]. Of the two cases, one was an infant who presented with hematemesis and the other was an adult on anticoagulation [3, 4].

Our case is a rare presentation of gastric hyperplastic polyp which resulted in massive gastrointestinal bleeding. Endoscopic measures did not control bleeding and finally surgical wedge resection helped in controlling bleeding, though the patient did not survive due to other reasons. Physicians encountering hyperplastic gastric polyps should be aware of this rare complication as the current endoscopic management options can prevent short-term and long-term complications.
